# Autism Spectrum Disorders: From Genotypes to Phenotypes

**DOI:** 10.3389/fnhum.2014.00914

**Published:** 2014-11-12

**Authors:** Valsamma Eapen, Raymond A. Clarke

**Affiliations:** ^1^School of Psychiatry, University of New South Wales and Academic Unit of Child Psychiatry, Ingham Institute, South West Sydney (AUCS), Liverpool Hospital, Sydney, NSW, Australia; ^2^Ingham Institute, School of Medicine, University of Western Sydney, Sydney, NSW, Australia

**Keywords:** Autism spectrum disorder, pathogenesis, etiology, genotype, phenotypic variability, review

Autism spectrum disorder (ASD) is appropriately named due to the broad variability or clinical heterogeneity, which in turn is linked to genetic heterogeneity. Such clinical heterogeneity is difficult to understand and difficult to treat under the auspices of a single disorder. The logical approach to this problem is to try to reduce the heterogeneity by stratifying ASD patients into smaller more homogeneous subgroups. However, this task is difficult as the stratification of ASD on the basis of behavioral differences would require clinicians to make evermore “new” categories of behavioral disorders when the core neurobiological deficits may be the same or similar. This difficulty is further amplified when the extreme genetic heterogeneity associated with ASD is considered and where some comparable genetic lesions are associated with different behavioral profiles and in some cases with different neuropsychiatric conditions. This Research Topic of Frontiers addresses these problems from a diversity of perspectives with the aim of clarifying these issues and promoting novel solutions.

The review by Eapen et al. (2013) sets the tone for this Research Topic of Frontiers by putting clinical heterogeneity in perspective with ASD genotypes, phenotypes, and potentially corresponding treatment outcomes necessitating the need to search for useful endophenotypes to help improve early intervention. The authors provide a perspective to the continuum of genetic variants (rare and common) present within the general population that has the potential to impact social-cognition and behavior. This also provides the ideal introduction to the genome-wide association study reported by Jones et al. (2013) that identified two ASD candidate genes (*PRKCB1* and *CBLN1*) from the general population with nominal association to autistic-like traits. *PRKCB1* (the gene encoding Protein kinase C-B1 involved in signal transduction and the regulation of gene expression) has been reported previously in linkage and association studies for ASD; while CBLN1 is just one of the many ligands for the neurexins – neuronal cell adhesion molecules involved in synaptogenesis and neural circuitry.

Neurexin mutations are common in ASD and all of the gene families encoding neurexin trans-synaptic ligands have been implicated previously in ASD. Together, the neurexins (NRXNs 1–4) and their different trans-synaptic ligands (CBLNs/GRIDs, LRRTMs, and NLGNs) are referred to collectively as the neurexin trans-synaptic connexus (NTSC). Here, Clarke and Eapen (2014) review the NTSC in detail as the basis for the molecular stratification of ASD. As common as NTSC mutations are in ASD, they are even more common in Tourette syndrome including recurrent disruptions of the intergenic region around the CBLN2 gene. NTSC mutations are also found in patients with Schizophrenia and intellectual disability. As such, Clarke et al. argue that the NTSC should represent the primary determinate for the molecular stratification of ASD and related neurodevelopmental disorders, from where characterization of the genetic architecture should provide a window for understanding how the NTSC and secondary variants function to specify behavior.

Recapitulating ASD models in rodents for mutations like those within the NTSC provide probably the best approach for improving drug development for ASD. The study by Argyropoulos et al. (2013) takes this one step further by describing the use of rodent models to identify and characterize endophenotypes that might be useful for the stratification of ASD patients as indicators of the biological pathways affected. This bottom-up approach is taken further by Unwin et al. (2013) in their study that suggests that the risk factors of “low birth weight” and “*in utero* exposure” to selective serotonin reuptake inhibitors give rise to some of the novel endophenotypes such as “sleep disturbance” in the pregnant mother and “gastrointestinal complaints in children with ASD” that could be useful for stratifying patients on the basis of cause and effect. Further, Billeci et al. (2013) have reviewed the use of advanced EEG techniques that has the potential to find distinctive patterns of abnormalities in ASD subjects, paving the way for the development of tailored intervention strategies. Similarly, the use of neuroimaging techniques to explore the heterogeneity in ASD is the focus of the review by Lenroot and Yeung (2013).

It appears that the phenotypic variability within ASD and the phenotypic overlap between ASD and other neurodevelopmental disorders such as Tourette syndrome, attention deficit hyperactivity disorder (ADHD), schizophrenia, language disorder, and intellectual disability could be due to the fact that the genes converge toward a core set of dysregulated biological processes that affect distinct neurodevelopmental pathways involved in synapse development/maintenance and circuitry formation through effects on neurogenesis, axon guidance in dendritic projections, and/or neuronal migration. Thus, defects in synaptic development can result in abnormal development across disorders and broad domains but yet carry distinct neurocognitive and behavioral profiles. The penetrance of the different comorbidities may in turn be related to the dose effects of gene abnormality or the timing of events when different neuronal regions and circuitry are being formed, as may be the influence of gender, intrauterine and perinatal events, epigenetics, and other environmental modulators. In this regard, accumulating evidence supports the notion that immune cells play important roles in normal brain function, outside of neuroinflammation. Voineagu and Eapen (2013) have reviewed recent data demonstrating the involvement of synaptic dysfunction and abnormal immune responses in ASD, and in particular the role of microglia in synaptic pruning during postnatal brain development, a period that coincides with the onset of ASD symptoms.

Given the multi modal and diverse origins of ASD, including the genetic as well as environmental modulation in the etiology, therapeutic interventions should also reflect such diversity. Furthermore, it seems that genetically mediated deficits and consequent functional impairments involve activity-dependent synapse development that depends on postnatal learning and experience. In this regard, the paper by Vivanti et al. (2013) suggests that intellectual disability in ASD might emerge as a consequence of severe social-communication deficits on the experience-dependent mechanisms underlying neurocognitive development. Such a model would predict that early intervention will prevent or reduce the risk of these deficits cascading into a trajectory toward full expression of the disorder by exploiting the neuronal maturation and brain plasticity. Thus, identifying homogeneous subgroups within ASD and matching appropriate interventions remain the key challenge for future research.

## Conflict of Interest Statement

The authors declare that the research was conducted in the absence of any commercial or financial relationships that could be construed as a potential conflict of interest.
